# Supplementary material for: Chromatin endogenous cleavage provides a global view of yeast RNA polymerase II transcription kinetics
Source: bioRxiv. 2024 Oct 10:2024.07.08.602535. Preprint. [Version 3] doi: 10.1101/2024.07.08.602535 (PMC11257477; doi:10.1101/2024.07.08.602535)
Supplement: Supplement 5 [file NIHPP2024.07.08.602535v3-supplement-5.pdf]

bioRxiv preprint doi: https://doi.org/10.1101/2024.07.08.602535; this version posted October 10, 2024. The copyright holder for this preprint (which was not certified by peer review) is the author/funder, who has granted bioRxiv a license to display the preprint in perpetuity. It is made available under aCC-BY 4.0 International license.

Figure 2 Supplement 1. RNAPII ChEC vs ChIP. (A) Gene plots displaying mean counts per million reads (CPM) from Rpb1 ChIP-seq (Vijjamarri et al., 2023a) or ChEC-seq2 with Rpb1-MN or Rpb3-MN over the *ILV1* and *GAL1-10* loci. A region spanning 1 kb upstream and downstream of each locus is displayed and arrows mark the transcription start site (TSS) and transcription end site (TES). Truncated arrows represent neighboring genes that continue outside of the displayed range. Plots are smoothed with a step size of 5 and window of 10. Signal from the Soluble MNase (sMNase) control is shown in black. **(B)** Metapromoter plots showing average signal flanking the transcriptional start site ± 250 bp from 150 genes with highest expression from STM and TFO classes (Rossi et al., 2021) and 84 repressed (Rep.) genes (Supplementary Table 1). **(C)** Correlation of nascent or total mRNA levels (measured by SLAM-seq) and either ChIP-seq (left) or ChEC-seq2 (right) signal over the indicated regions of each gene. Spearman correlation coefficients for each are shown. **(D)** Nacent RNA levels from SLAM-seq for each class of genes from Rossi et al., 2021. **(E)** Metasite plot of 597 expressed, mRNA-encoding genes aligned by their TATA sequence (genes listed in Supplementary Table 1; Rhee and Pugh, 2012). Average signal from sMNase grown in rich medium is plotted. A window spanning ± 250 bp around the TATA sequence, with the TSS to the right, is shown. The location of the TATA sequence is indicated with a grey bar. The range encompassing TSSs is indicated and the rectangle below the plot designates the approximate location of the CDS. **(F)** Predicted occupancy of RNAPII based on a range of promoter dwell times (0.1 - 2 s), elongation rates (1000 - 3000 bp/min) and termination times (1 - 2 s). The transcribed region is 1200 bp divided into 10 x 120bp bins, flanked by an upstream promoter bin and downstream terminator bin. RNAPII occupancy was simulated using a minimal stochastic model. RNAPII was assumed to be immediately present at the promoter and progressed to the transcript region with a rate inverse to the promoter dwell time. It then progressed along a 1200 bp coding region with the indicated elongation rate and terminated transcription with a rate inverse to the terminator dwell time.

Figure 2 Supplement 2. ChIP-seq against Rpb1 vs. Rpb1-MN. (A) Metagene plots showing the ratio of signal between IP and Input fractions (IP/Input) over subsets of genes with distinct expression levels and mechanisms of regulation. The average signal from 150 genes with highest expression from STM and TFO classes (Rossi et al., 2021) and 84 repressed genes is plotted (genes listed in Supplementary Table 1). A length-normalized transcript (arrow), 1 kb upstream of the TSS, and 1 kb downstream of the TES is shown. Rpb1 ChIP-seq (left; blue), Rpb1-MN ChIP-seq (right; purple). **(B)** Correlation of ChIP-seq against Rpb1 vs. Rpb1-MN. Signal (CPM) over the indicated regions of each gene are compared. Spearman correlation coefficients for each are shown. The average of three biological replicates is shown in (A) and (B).

bioRxiv preprint doi: https://doi.org/10.1101/2024.07.08.602535; this version posted October 10, 2024. The copyright holder for this preprint (which was not certified by peer review) is the author/funder, who has granted bioRxiv a license to display the preprint in perpetuity. It is made available under aCC-BY 4.0 International license.

Figure 3 Figure supplement 1. Mintbody-directed ChEC. (A) Genomic DNA isolated from strains expressing Ser5p-MN (JVY305) and Ser2p-MN (JVY302) was analyzed on a TapeStation 4150. MNase

was activated and cleavage proceeded for 30 seconds (red), 60 seconds (green), or 120 seconds (blue). Genomic DNA isolated from cells where no cleavage occurred is shown in black. Note: absolute determination of molecular weight above 50 kb is not possible with this assay and is shown here to highlight relative changes in molecular weight between samples. **(B)** Chemiluminescent western blot of strains expressing Mintbody-MNase constructs specific to Ser2 phosphorylation (a-Ser2p-MN, JVY302) or Ser5 phosphorylation (α-Ser5p-MN, JVY305) of the CTD of RNAPII. Strains expressing each construct on the *kin28is* background are also shown (α-Ser2p-MN, JVY314; α-Ser5p-MN, JVY317). **(C)** The relative enrichment at UAS, promoter, transcript, and 3'UTR regions was calculated and normalized by region length for each gene. The average from all genes in each group is plotted. Error bars represent the standard error of the mean between three biological replicates.

Figure 5 Supplement 1. Growth effect of CMK treatment in wild type and *kin28is* cells. (A) OD_{600} of *kin28is* strain grown at 30°C in synthetic complete medium with the indicated concentrations of CMK. The average ± standard deviation is plotted.

bioRxiv preprint doi: https://doi.org/10.1101/2024.07.08.602535; this version posted October 10, 2024. The copyright holder for this preprint (which was not certified by peer review) is the author/funder, who has granted bioRxiv a license to display the preprint in perpetuity. It is made available under aCC-BY 4.0 International license.

Figure 6 Figure supplement 1. Parameter fitting of unknown transcription rates. Rates with no known value were fit to RNAPII occupancies from either ChEC-seq2 or ChIP-seq data using a grid search (see Methods). **(A-C)** For STM model, rates k_2, k_{-2}, and k_4 were explored in the range [0, 0.2] and k_{-3} in the range [0, 0.03]. For TFO model, only rates k_{-3}, and k_4 were fit, in the same range. **(A)** Distribution of cosine similarity for the model ensemble when fit to ChEC-seq2 data. Cosine similarity of 1 indicates perfect alignment, 0 indicates no correlation, and -1 indicates perfect inverse alignment. **(B)** Distribution of cosine similarity for the model ensemble when fit to ChIP-seq data. **(C)** Rate combinations that fit the empirical data ChEC-seq2 data (Rpb1-MN). This resulted in 789 rate combinations for the STM model and 371 rate combinations for the TFO model. **(D)** No rate combinations resulted in a satisfactory fit to the empirical ChIP-seq data (Rpb1). Instead, an equal number of rate combinations (best-fit) as shown in (**C**) is displayed. **(E)** In an attempt to identify rates that fit the RNAPII enrichment seen by ChIP-seq, we used the promotor-recruitment model (TFO genes) and loosened previously fixed rates k_5 and k_7 and expanded the search range for k_4 while fixing k_{-3}. Published rates for k_5 and k_7 are displayed in red on the axes. Range from parameter fit k_4 from ChEC-seq2 data (C) is shown in orange on the k_4 axis. The range of values for k_4, k_5, and k_7 that fit the ChIP-seq data are shown in the table (Functional Range). In the idealized case k_{-3} = 0 and k_4 is instantaneous then k_7 should be equal to the product of k_6 and the ratio between the average occupancy given by ChIP in the coding region and terminator of the gene (approximately 0.14 s^{-1}), and k_5 should be equal to the product of k_6 and the ratio between the average occupancy given by ChIP in the coding region and the terminator of the gene (approximately 0.2 s^{-1}). The functional ranges shown agree with this, as rate k_5 is bounded below by the idealized approximation, and rate k_7 is centered around its idealized approximation. **(F)** The average Rpb1 signal (purple) from ChIP-seq over the indicated regions from TFO-class genes that are expressed in SDC. RNAPII enrichment resulting from rate combinations shown in (E) were modeled in combination with fixed rates from the literature shown in Table 1. UAS, Promoter, and 3'UTR were represented by a single 120 bp bin and the transcript region was composed of 10 sequential bins to represent a 1200 bp transcript. The average predicted occupancy for RNAPII over each region from the models (*i.e.* sets of rates) that best matched the empirical data are shown (see Methods). For Rpb1 ChIP-seq, 55 rate-combinations from the promoter model fit the empirical data from TFO-class genes. Empirical and model outcomes were compared for each gene region with a Student's t-test, which reported no significant differences ($p > 0.05$).

bioRxiv preprint doi: https://doi.org/10.1101/2024.07.08.602535; this version posted October 10, 2024. The copyright holder for this preprint (which was not certified by peer review) is the author/funder, who has granted bioRxiv a license to display the preprint in perpetuity. It is made available under aCC-BY 4.0 International license.

Figure 7 Figure supplement 1. Parameter fitting of unknown transcription rates in UAS-recruitment model for Gcn4 target genes. Rates with no known value were parameter fit using a grid search (see Methods). We used the UAS-recruitment model and explored rates k_2, k_{-2}, and k_4 in the range [0, 0.2] and k_{-3} in the range [0, 0.03]. Rate combinations that fit the Rpb1-MN ChEC-seq2 data from 287 Gcn4-target genes under amino acid starvation conditions. The fitting procedure resulted in 1057 rate combinations that fit the empirical data.

bioRxiv preprint doi: https://doi.org/10.1101/2024.07.08.602535; this version posted October 10, 2024. The copyright holder for this preprint (which was not certified by peer review) is the author/funder, who has granted bioRxiv a license to display the preprint in perpetuity. It is made available under aCC-BY 4.0 International license.

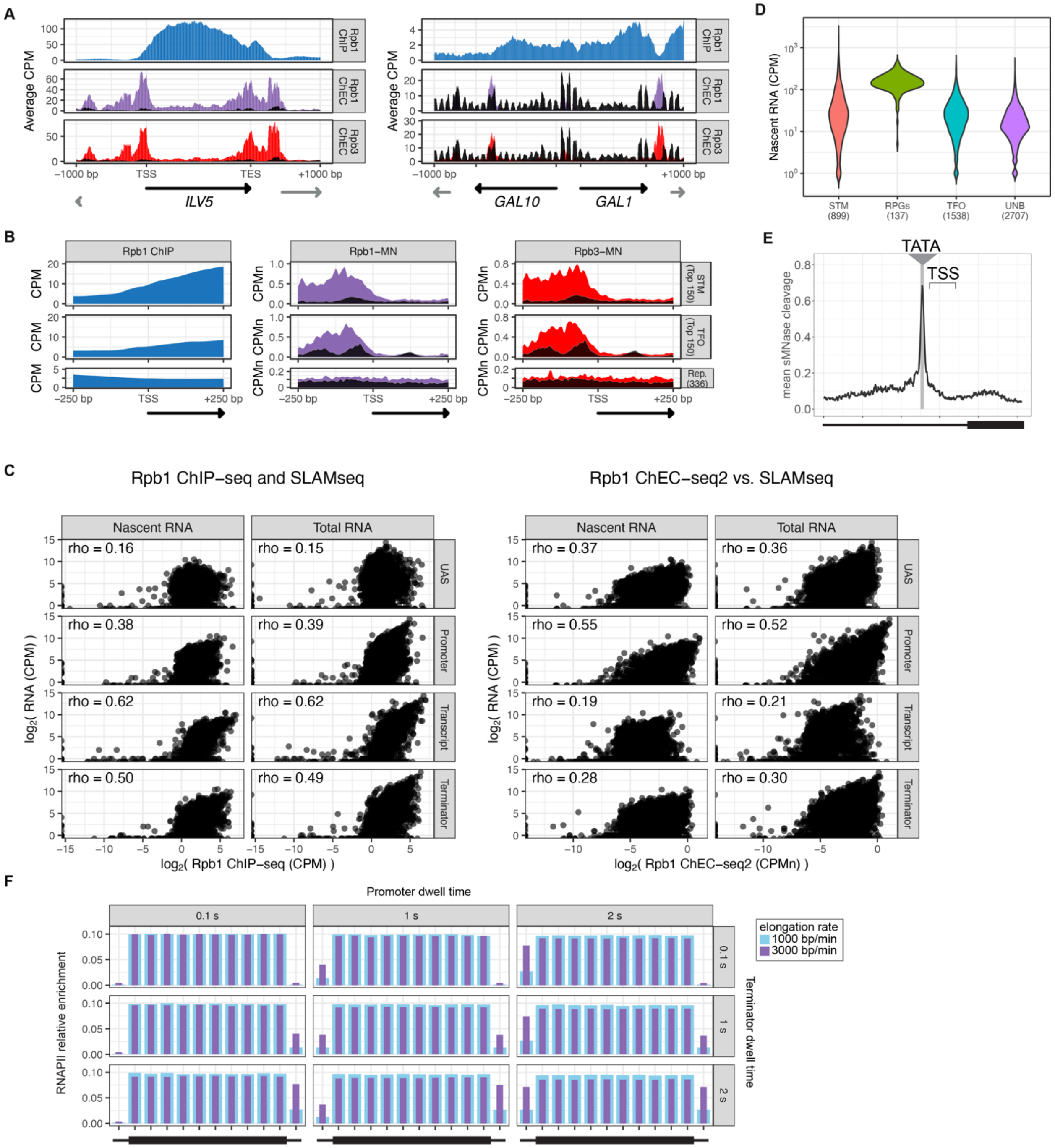

VanBelzen Figure 2 - supplement 1

bioRxiv preprint doi: https://doi.org/10.1101/2024.07.08.602535; this version posted October 10, 2024. The copyright holder for this preprint (which was not certified by peer review) is the author/funder, who has granted bioRxiv a license to display the preprint in perpetuity. It is made available under aCC-BY 4.0 International license.

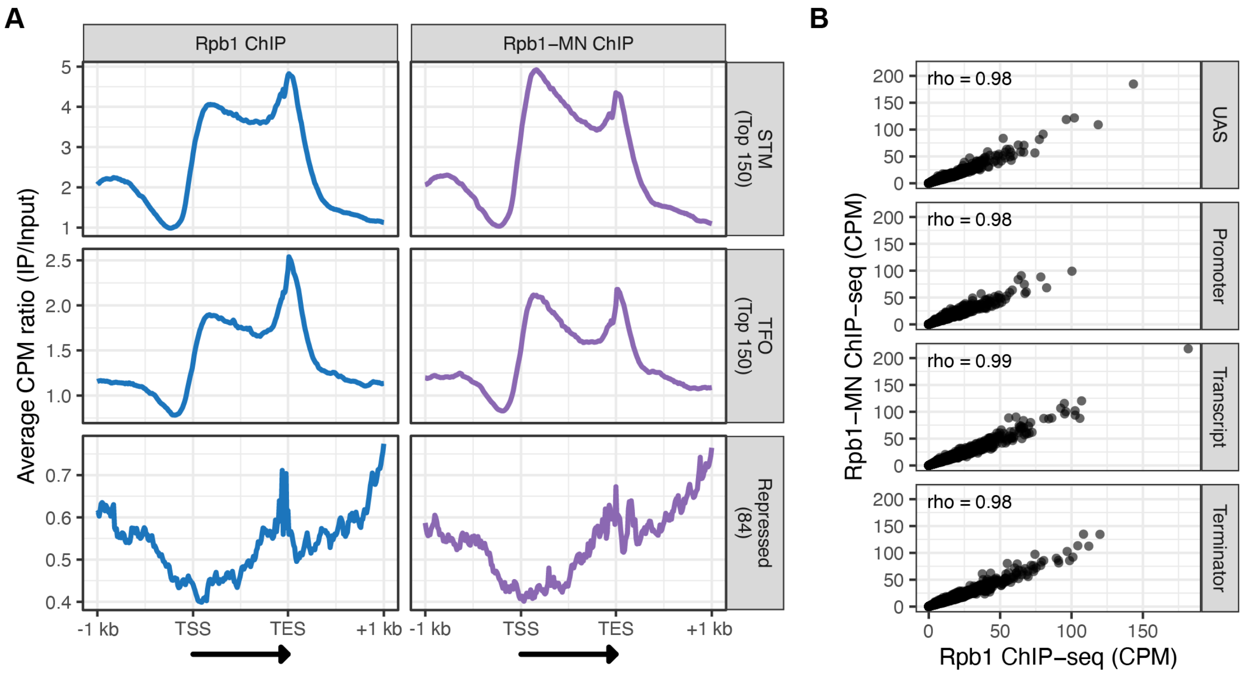

VanBelzen Figure 2 - supplement 2

bioRxiv preprint doi: https://doi.org/10.1101/2024.07.08.602535; this version posted October 10, 2024. The copyright holder for this preprint (which was not certified by peer review) is the author/funder, who has granted bioRxiv a license to display the preprint in perpetuity. It is made available under aCC-BY 4.0 International license.

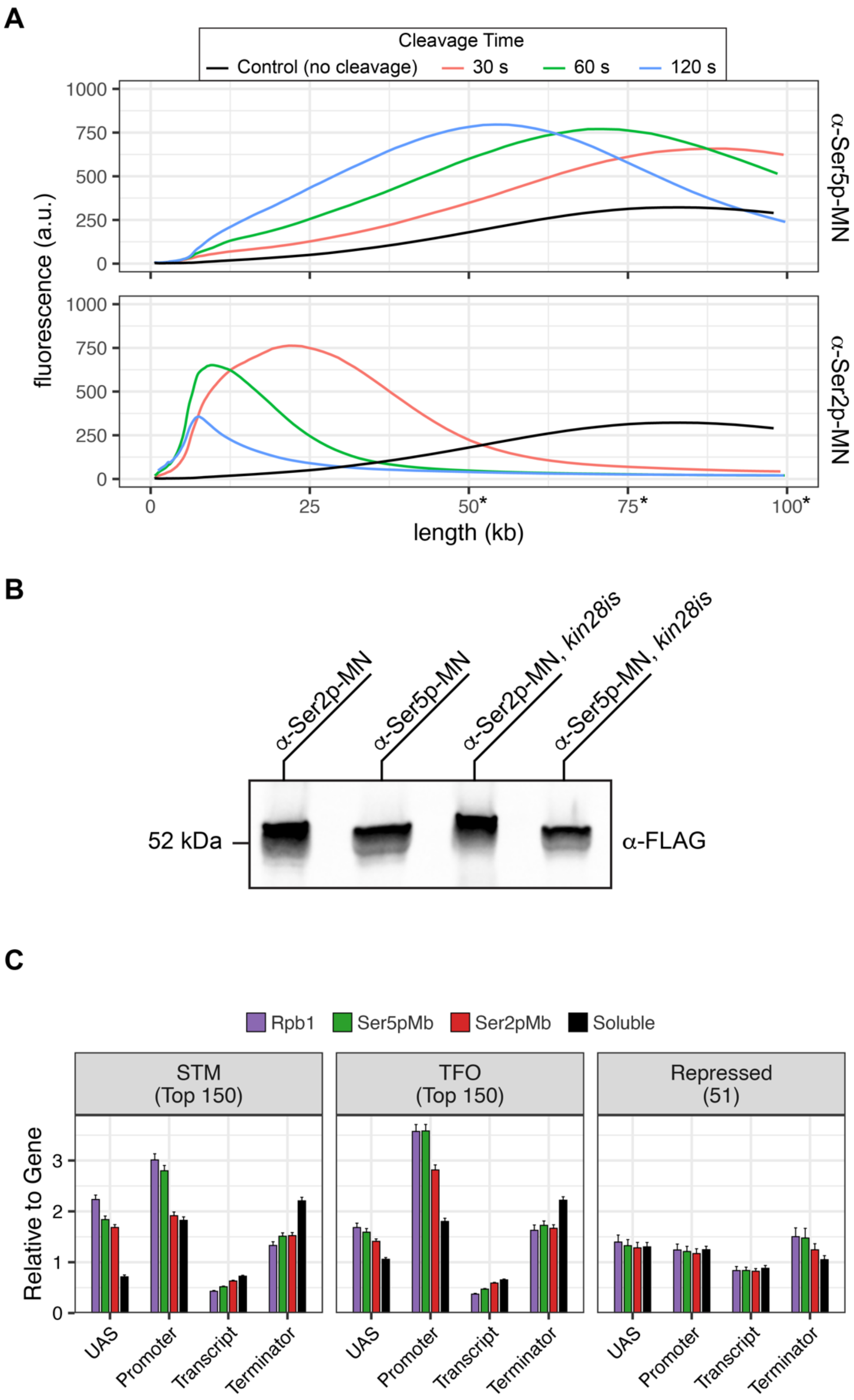

VanBelzen Figure 3 - supplement 1

bioRxiv preprint doi: https://doi.org/10.1101/2024.07.08.602535; this version posted October 10, 2024. The copyright holder for this preprint (which was not certified by peer review) is the author/funder, who has granted bioRxiv a license to display the preprint in perpetuity. It is made available under aCC-BY 4.0 International license.

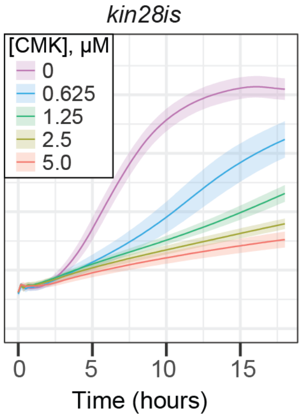

VanBelzen et al., Figure 5 - supplement 1

bioRxiv preprint doi: https://doi.org/10.1101/2024.07.08.602535; this version posted October 10, 2024. The copyright holder for this preprint (which was not certified by peer review) is the author/funder, who has granted bioRxiv a license to display the preprint in perpetuity. It is made available under aCC-BY 4.0 International license.

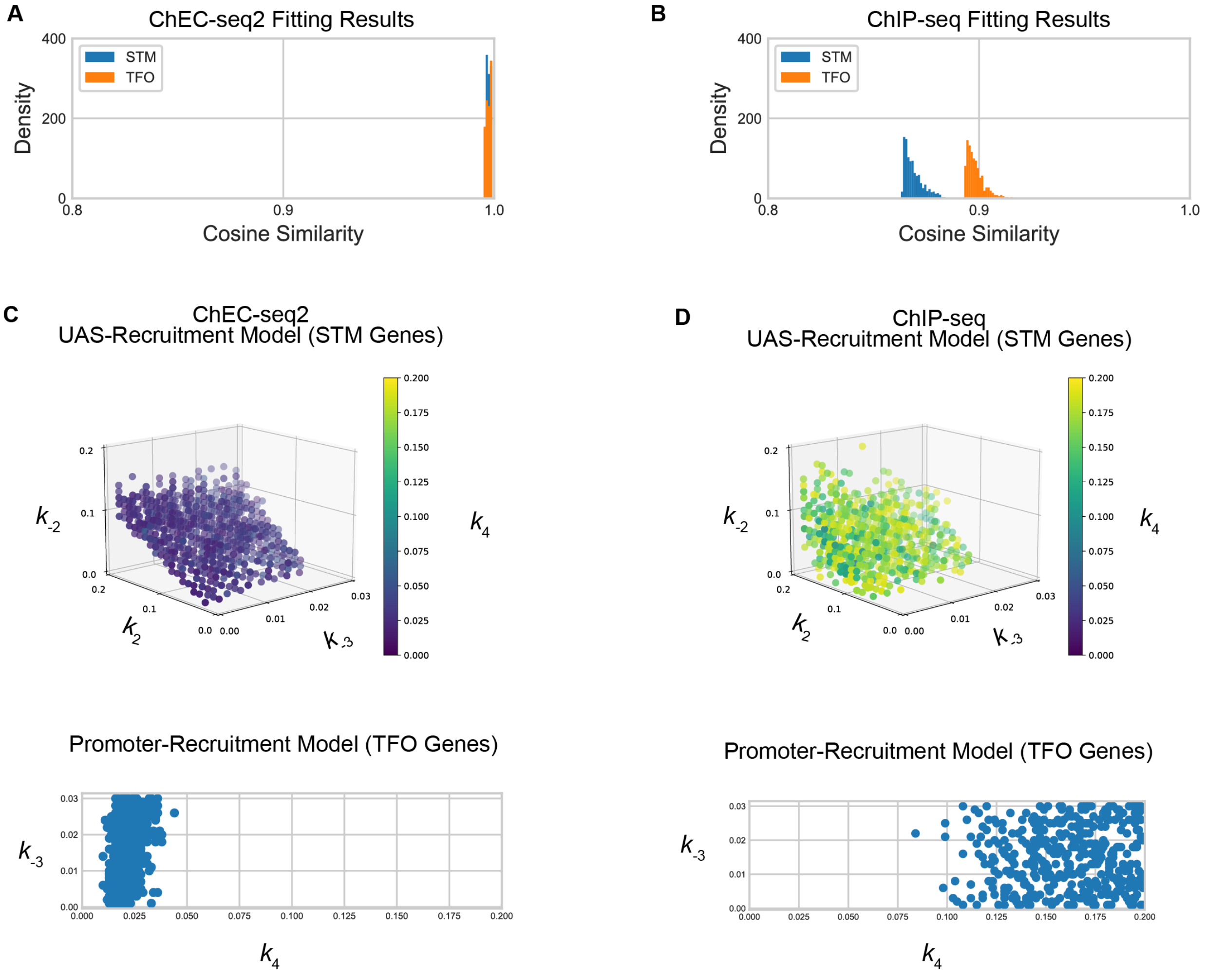

E

ChIP-seq
Expanded Parameter Range (TFO Genes)

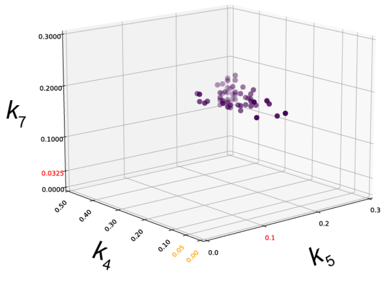

Rate	Fixed Rate	Functional Range
$k_{3,Pro}$	0.002/s	-
k_{-3}	0.003/s	-
k_4	-	0.28 - 0.5/s
k_5	-	0.23 - 0.3/s
k_6	1 kb/min	-
k_7	-	0.11 - 0.17/s

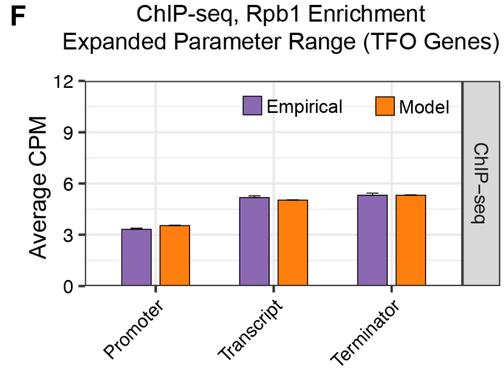

VanBelzen et al., Figure 6 supplement 1

bioRxiv preprint doi: https://doi.org/10.1101/2024.07.08.602535; this version posted October 10, 2024. The copyright holder for this preprint (which was not certified by peer review) is the author/funder, who has granted bioRxiv a license to display the preprint in perpetuity. It is made available under aCC-BY 4.0 International license.

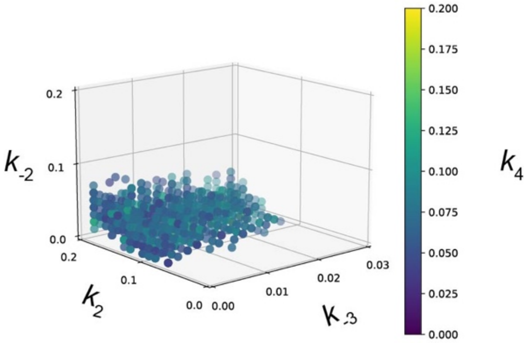

VanBelzen et al., Figure 7 - supplement 1
